# Manifestations of HIV stigma and their impact on retention in care for people transitioning from prisons to communities

**DOI:** 10.1186/s40352-017-0054-1

**Published:** 2017-06-06

**Authors:** Rebecca Kemnitz, Theresa C. Kuehl, Karli R. Hochstatter, Emily Barker, Anna Corey, Elizabeth A. Jacobs, Michael D. Repplinger, William J. Ehlenbach, David W. Seal, James M. Sosman, Ryan P. Westergaard

**Affiliations:** 1 University of Wisconsin Madison School of Medicine and Public Health, Madison, WI USA; 2 Tulane University School of Public Health, New Orleans, LA USA

**Keywords:** Human immunodeficiency virus, Antiretroviral therapy, Incarceration, Stigma, Social support

## Abstract

**Background:**

While most people living with HIV who are incarcerated in United States receive appropriate HIV care while they are in prison, interruptions in antiretroviral therapy and virologic failure are extremely common after they are released. The purpose of this study was to describe whether and how HIV stigma influences continuity of care for people living with HIV while they transition from prison to community settings.

**Methods:**

We conducted semi-structured, telephone-based interviews with 32 adults who received HIV care while residing in a Wisconsin state prison, followed by a second interview 6 months after they returned to their home community. Interview transcripts were analyzed by an interdisciplinary research team using conventional content analysis. We identified themes based on commonly-reported experiences that were characterized as internalized stigma, perceived stigma, vicarious stigma, or enacted stigma.

**Results:**

All four forms of HIV stigma appeared to negatively influence participants’ engagement in community-based HIV care. Mechanisms described by participants included care avoidance due to concerns about HIV status disclosure and symptoms of depression and anxiety caused by internalized stigma. Supportive social relationships with clinic staff, professional case managers and supportive peers appeared to mitigate the impact of HIV stigma by increasing motivation for treatment adherence.

**Conclusions:**

HIV stigma is manifest in several different forms by people living with HIV who were recently incarcerated, and are perceived by patients to negatively influence their desire and ability to engage in HIV care. By being cognizant of the pervasive influence of HIV stigma on the lives of criminal justice involved adults, HIV care providers and clinical support staff can ameliorate important barriers to optimal HIV care for a vulnerable group of patients.

## Background

The U.S. prison population carries an excess burden of HIV/AIDS compared to the general population (Maruschak, 2012). An estimated 1.5% of incarcerated U.S. adults are living with HIV, which is approximately three times greater than the prevalence of the general population. Antiretroviral therapy is effectively administered in most prison settings, and those receiving HIV care in prison tend to have good treatment outcomes (Meyer et al., 2014a; Springer, Friedland, Doros, Pesanti, & Altice, 2007). A major challenge, however, is ensuring that HIV treatment continues without interruption after release. The process of community re-entry poses significant challenges to managing HIV, including risk of substance use relapse, poor access to mental health care, and lack of employment, housing, transportation, and education (Brinkley-Rubinstein, 2013; Small, Wood, Betteridge, Montaner, & Kerr, 2009). Engagement in HIV care, including attendance at scheduled clinic appointments and adherence to antiretroviral therapy tends to be inconsistent during the re-entry period, leading to frequent lapses in treatment and virologic failure (Baillargeon et al., 2009; Meyer et al., 2014a; Springer et al., 2004).

Stigma associated with one’s positive HIV status is one of the numerous individual-level, social, and structural factors that negatively influences the health of formerly incarcerated people. As defined by Goffman (1963), stigma is “an attribute that links a person to an undesirable stereotype, leading other people to reduce the bearer from a whole and usual person to a tainted, discounted one” (p. 3). HIV-related stigma was conceptualized by Parker and Aggleton (2003) as both a manifestation and a driver of social inequality, representing a structural issue that leads to health disparities. Evidence that HIV stigma shapes the ways people living with HIV engage in care is common in the literature. In a meta-analysis of 75 studies from 32 countries, Katz et al. (2013) found that HIV stigma was consistently associated with poorer antiretroviral adherence. Research delineating mechanisms by which stigma leads to poor HIV treatment outcomes is less common, but several formative studies suggest its effects are multi-faceted and complex. A qualitative study by Haley et al. (2014) suggested that HIV stigma negatively influenced many aspects of respondents’ lives, including acting as a trigger for substance use and impairing one’s ability to prioritize HIV care. Brinkley-Rubinstein (2015) described intersecting influences of racial stigma, HIV stigma and incarceration stigma, which drive people to disengage from usual sources of health care.

To contribute to our growing understanding of how the experience of HIV stigma influences the HIV-related health of criminal justice-involved adults, we conducted a qualitative analysis of interviews with 32 individuals who received HIV care while in prison and were subsequently released. Through paired interviews scheduled several weeks before and six months after they were released, we aimed to study in-depth the experiences of people who engaged in HIV care both while incarcerated and in the community. This qualitative analysis is a component of a larger, mixed-methods study that longitudinally examines the HIV treatment outcomes of a cohort of patients involved with the criminal justice system. The overall aim of the parent study is to identify modifiable risk factors for suboptimal engagement in HIV care and lapses in antiretroviral adherence during the transition from prisons to communities.

## Methods

### Study setting and participants

The Wisconsin Transitions Study is an ongoing observational, prospective cohort study initiated in 2013 to comprehensively evaluate barriers to engagement in HIV care after release from prison. While incarcerated, all participants receive HIV care at the University of Wisconsin HIV/AIDS Comprehensive Care Clinic, the sole provider of antiretroviral treatment for the Wisconsin Department of Corrections (DOC). Inclusion criteria specify that participants must be 18 years of age or older, English-speaking, HIV-positive and intend to receive HIV care in the community upon release. Informed consent, participant demographic information, and contact information necessary for ensuring study follow-up after release are obtained at an in-person clinic visit. Subsequent study assessments are conducted via telephone over an unmonitored line in the Health Services Unit of the participant’s prison facility.

The first 2 years of this 5-year study involved intensive data collection using numerous modalities, both before and after release from prison. All eligible DOC patients receiving HIV care were recruited for the study, and those expecting to be released during the first phase were invited to complete monthly questionnaires and a longer post-release interview during the first six months after their release date. To encourage follow-up in the study after release, participants were given the option to receive a cellular phone with unlimited voice and text for the duration of the study or monetary compensation of $50 per month, with the value of either option totaling approximately $400 per participant. The analysis presented in this paper uses data derived from the pre-release and post-release interviews conducted among the participants enrolled during the first phase of the study. A sample size of 20–30 participants was expected to be necessary for the qualitative component of the study in order to achieve theme saturation. Quantitative data obtained through questionnaires, electronic health records, and HIV surveillance systems are still being collected, and will be analyzed and disseminated in future reports.

### Data collection

Semi-structured interview guides were developed with open-ended questions that were informed by the situated information-motivation-behavioral skills (sIMB) framework of behavioral determinants of retention in HIV care (Amico, 2011). The main outcome of interest was continuity of HIV care in the community after release from prison. Participants were invited to complete two interviews: the first interview occurred within one month prior to release and the follow-up interview approximately six months after the release date. The pre-release interview provided a baseline understanding of the participants’ experiences with HIV care prior to and during the current incarceration period and anticipated barriers to HIV care continuity upon release. The post-release interviews were designed to provide insight into participants’ experiences transitioning to community care after release. Full text of the interview guides used for this study is presented in the Appendix.

Each participant was asked the same initial questions, with follow-up questions and probes informed by their initial responses. A research assistant with a background in social work and additional training in qualitative research methods conducted all of the pre-release and post-release interviews. Initial questions asked during the pre-release interview included “Tell me about your priorities for your first few months after going home” and “How will these affect your HIV medical care?” A typical question at the post-release interview was “Tell me about your transition from HIV care in prison to HIV care in the community.” Using these questions and subsequent probes for further explanations or clarification of participants’ responses, we sought to understand participants’ attitudes and motivation to seek HIV care and how these factors shaped their care-seeking behavior. Interviews lasted between 30 and 60 min, were digitally audio-recorded directly from the telephone and later professionally transcribed. The research assistant reviewed audio recordings and collaborated with the transcriptionist to ensure accuracy of the transcripts, responding to queries related to recorded speech that was difficult to understand. Once verified, transcripts were imported into a data analysis software package, NVivo Version 10 (QRS International, Doncaster, Australia).

### Data analysis

The first 5 de-identified interview transcripts were read and discussed by an interdisciplinary research team to develop familiarity with the early data and agree upon an analytic approach. The team was comprised of a social psychologist, three physicians, two medical students and two research assistants with training in social work and public health. The qualitative technique used was conventional content analysis (Hsieh & Shannon, 2005). A standard codebook was developed and used by the research team to identify common themes that appeared in the text of the interview transcripts.

The role of HIV stigma in continuity of HIV care was not selected as a focus of the interviews a priori by the research team, but rather emerged as an important and pervasive theme from the experiences reported by early participants. To explore stigma-related content emerging from our early analysis, we identified passages of text appearing to connote experiences of stigma and developed supplemental stigma-related codes to be used data analysis. The supplemental codebook divided stigma into the four types of stigma established by Churcher (2013), and included the main headings of internalized, perceived, enacted, and vicarious stigma. Each main heading was further subdivided into 5–10 smaller categories to more specifically characterize the context of the stigma reported. For example, perceived stigma could reflect specific interpersonal encounters or general perceptions about public opinions, and therefore multiple codes were developed to capture these distinctions.

All interviews were coded individually by at least two members of the research team, who engaged in an iterative process of coding to achieve theme saturation (Morse, 1995). Any discrepancies in codes were resolved via consensus, referring back to the transcripts and discussing codes until there was 100% agreement on designated codes. When possible, interviews were analyzed as pairs, representing the pre-release and post-release interview from each individual participant. The research team finally met to reach consensus about the most important themes occurring throughout the dataset and selected illustrative quotes to aid in the presentation of the findings.

## Results

### Participants

Beginning in October 2013, a research assistant screened all patients referred for HIV care by the Wisconsin DOC for eligibility in the study. To date, 131 of 185 eligible individuals (71%) have been approached during a clinic visit, and of these, 128 (98%) provided informed consent to participate. Of the 3 refusals, 1 individual stated he did not have time to participate because of a work-release program, 1 did not provide a reason for refusal, and 1 stated “I do not want to talk about HIV any more than I have to.” The first 32 participants who were released from prison during the intensive data collection phase were interviewed. The sample size was slightly larger than expected because a number of participants were unavailable to complete the planned post-release interview. Twenty-three participants (72%) completed both a pre-release and post-release interview, 8 completed a pre-release interview only, and 1 completed a post-release interview only. Of the 8 participants who failed to complete the post-release interview, 6 were determined to have been re-incarcerated, 1 was discovered to have sold the study phone, and 1 was simply lost to follow-up.

The mean participant age was 41 years (range 19 to 68). Twenty-seven participants were male, three were female, and two were transgender women. Twenty-two participants were African American, eight were White/non-Hispanic, and two self-identified as White and Hispanic/Latino. Nine of the male participants self-identified as men who have sex with men (MSM). Nine participants reported a history of injection drug use. Two declined to answer about previous injection drug use.

### Experiences of HIV stigma

All four types of stigma were experienced by numerous participants. Internalized stigma was reported most frequently; this code appeared 222 times, in 41 of the 55 transcripts analyzed. Vicarious stigma was mentioned least frequently (3 codes in 3 transcripts). In many cases, multiple forms of stigma were reported to co-occur: Participants who experienced externalized stigma often also reported high levels of internalized stigma. In nearly all cases, reported stigma was directly linked by the participants as reasons for poor adherence to care, including missed appointments or missed doses of medication. Descriptions of perceived and experienced HIV stigma were not substantially different when taken from pre-release versus post-release interviews. Most participants had been incarcerated multiple times in the past, and recounted their prior transitions in HIV care similarly in the pre-release and post-release interviews.

#### Internalized stigma

Nearly all participants (*n* = 30) reported some form of internalized stigma, represented as status disclosure concerns, expressions of shame related to HIV risk behaviors, or negatively connoted internal representations of HIV. Several participants cited adverse emotional responses to diagnosis as a reason for missed appointments. One participant reported:

*I would just get depressed, so sometimes I would just make up excuses, just like, oh, I’m just tired, and, because I really didn’t want to face what I had to do because it was just, it was just another reminder in my face that, you know, what I carry.* (24 year-old transgender woman, pre-release interview)

The most commonly reported instances of internalized stigma stemmed from concerns about disclosure of HIV status. In some cases, prior negative experiences of disclosing their HIV status to others contributed to feelings of shame and anxiety. These emotional responses influenced future behaviors and social interactions, at times leading to avoidance of situations in which the confidentiality of their HIV status might be threatened. Fear of disclosure was a frequently cited barrier to keeping appointments with HIV care providers. Discomfort related to the public nature of clinic waiting rooms was a common example of this:

*I don’t want to have to go somewhere where I don’t feel comfortable . . . I’m going to walk into this room and I know why everybody there, and everybody know why I’m there. I guess I can say I do privy myself of what people think of me, you know, how did he get it, what they’re thinking in their head when I walk through the door. Those are a lot of questions in the back of my head that I’m thinking, you know, I guess I’m a self-preserved type of person.* (44 year-old man, pre-release interview)

Fearing disclosure of one’s HIV status to family or friends was linked to poor medication adherence. Participants avoided disclosure by not bringing pill cases or antiretroviral drugs with them when they were around friends, leading them to miss doses if they were away from home for an extended period. One participant described this type of situation while living with the sister of a former partner.

*So they were always curious about me, so when [HIV care organization] dropped off those meds, she Googled it on her phone and saw that the pharmacy was part of the [HIV care organization] network, and basically she just tried to talk to me about it, but I found it offensive because I just, I didn’t want her to know. That wasn’t her business. So, I stopped taking the meds.* (44 year-old man, pre-release interview)

Placement in a Transitional Living Program (“halfway house”) was particularly problematic for several participants because the dormitory-style residence offered limited privacy.

*It’s easier for me to just carry the pills in my bag versus I got the bottles that they came in with the names on the bottles, they run into the wrong person so there’s somebody being nosy going through your stuff and they don’t know what this is and then they Google the name and then there go everything that the pill is.* (37 year-old man, post-release interview)

#### Perceived stigma

Perceived stigma refers to the anticipated responses of others to the participants’ HIV status and was the second most common reported type of stigma by study participants (*n* = 21). Reports of perceived stigma were classified as social reactions to HIV status, interpersonal experiences surrounding HIV, and perceived public attitudes about HIV. In the prison setting, this type of stigma could manifest as hypervigilance and anxiety due to participants’ fears of social sanctions or even violence resulting from disclosure of their HIV status.

*What I mean by keeping it hidden is, you know, holding it in and not letting others know about it or what they would think about me or what they would say about me or, you know, the kind of treatment that I would get if [other incarcerated men] would, you know, try to kill me or beat me up or, you know, just the derogatory name-calling, everything.* (44 year-old man, pre-release interview)

After release, participants continued to fear disclosure of their HIV status to peers, criminal justice staff (e.g. probation or parole officers), and even close contacts. Engagement in HIV care and antiretroviral adherence could be influenced by stigma, even if participants were not actually treated in a negative or harmful way.

*Basically I put my life on hold as far as medications and stuff because I was thinking about what other people say about me.* (43 year-old man, post-release interview)

Participants described others’ responses to disclosure of their status as expressions of pity or fear. Participants endorsed perceptions of public disapproval of HIV risk behaviors and judgment of people living with HIV as being personally responsible for their illness.

*Within like a year, I knew that something had to be done about it in my area, my community, because everybody was judging. There’s no reason to judge. It’s like, why would you judge someone like that? If you don’t judge someone with diabetes or leukemia or anything else, why would you judge somebody with HIV?* (61 year-old man, pre-release interview).

Participants who reported higher levels of perceived stigma commonly also reported high levels of internalized stigma and expressed negative attitudes about their HIV status. Negative attitudes, in turn, often corresponded with lower apparent motivation to engage in care seeking behaviors and avoidance of people and locations associated with HIV services. Unpleasant thoughts and experiences related to HIV were also described as triggers for unhealthy behaviors such as drug and alcohol misuse.

*You know, they were just ignorant to the disease as much as I was or anybody else was back then, so now I understand that. But it hurt me a lot, kind of turned me away from my family as far as, you know, resentment and, when I did think about all of that together, I just wanted to just drown it out, and I do drugs to drown it out.* (51 year-old man, pre-release interview)

#### Enacted stigma

Enacted stigma, or specific lived experiences perceived to reflect stigmatization of HIV, were described by several participants (*n* = 9) as a cause of psychological distress and served to reinforce the internalization of HIV stigma. Drawing distinctions between perceived stigma and enacted stigma is difficult in this study -- all the data derive from participants’ self-report and cannot be verified by other sources. Despite this inherent limitation, we identified numerous examples of participants observing others’ behavior that appeared to be driven by HIV stigma.

*[My parole officer] knows about my HIV status. And she never said anything, but I can tell her actions, the way she really treat me, you know, as far as [not] shaking my hand and some, it don’t really bother me, but I know she, either she’s not educated about HIV or she has got an attitude toward people with HIV, I don’t know.* (64 year old man, post-release interview)

Common examples of enacted stigma were instances of refusal to touch, hug, or shake hands with an HIV-positive individual, isolation behavior, or throwing away dishes or silverware used by the participant. Participants discussed losing friends, being rejected by family, and being rejected from social situations or gatherings as a result of their HIV status.

*I had told [my daughter’s mother] that I was sick and that her daughter was playing by me and she told me not to touch her daughter, and I . . . I stood there in shock. I’m like, what are you talking about? She’s like, you’re sick so I don’t want you to touch my daughter and, you know, that kind of hurt me, you know. And I felt like, dang, you know, I’m sick, so everybody’s looking at me different, you know.* (28 year-old man, pre-release interview)

#### Vicarious stigma

Vicarious stigma, referring to stories or events of HIV stigma witnessed by the participant, was mentioned by only three participants. A participant’s godfather was HIV positive and discussed the stigma he faced, shaping the way that the participant anticipated stigma he might face. He states:

*My godfather, he used to tell me a lot of things because he got his HIV through a blood transfusion, and he used to tell me that people would treat him different. People never wanted to sit by him. People never wanted to, you know, be around him and they didn’t want to talk to him for the simple fact he was HIV positive.* (28 year-old man, pre-release interview)

The other 2 participants’ reporting the experience of vicarious stigma discussed the general tendency for people around them to speak negatively about people living with HIV. Participants exposed to vicarious stigma frequently described experiences of internalized stigma as well. They reported that exposure to others’ negative HIV disclosure experiences caused them to anticipate negative or discriminatory responses to disclosure of their own HIV status.

### Social support mitigates the effects of HIV stigma

Participants described receiving beneficial social support from numerous sources, including family or friends, HIV care providers, and case managers. Family and friends provided mixed levels of support. Although in some cases, family and friends reinforced feelings of stigmatization, they frequently played a positive role in participants’ care. Supportive family and peers often reminded participants to take medication, provided transportation to appointments, or provided a stable home environment where medication could be stored and integrated into a daily routine. If family members accepted an individual’s status, that individual was more likely to accept their status, manage their HIV, and adhere to treatment. One participant described the role his friend’s acceptance plays in his keeping medical appointments after he was released:

*It means, staying on track means to, you know, go to my appointments, you know, then like I was telling you that, you know, the friend that I’m talking about she is understanding, so do a lot of talking about, you know, her situation. So she probably still is the only one that really accept me.* (42 year-old man, post-release interview)

Additionally, participants reported positive relationships with HIV care providers that mitigated the internalization of HIV stigma. Many participants discussed their care providers as the first people to help them accept their diagnosis, often through normalization techniques that demonstrated HIV-positive individuals living long and generally healthy lives after receiving their HIV diagnosis. When care was established and the relationship built, participants reported fewer concerns regarding status disclosure or discomfort with the clinic waiting room. Participants responded positively when asked about their clinic experience, citing good relationships with their provider and the clinic staff. For example, one participant reported arriving early to chat with a front desk staff member with whom he had developed a positive relationship. In spite of disclosure concerns expressed by many participants, this type of relationship with clinic staff encouraged participants to continue to receive care and attend appointments.

During the study period, several participants received additional services through a federally funded demonstration project that was implemented by the Wisconsin Division of Public Health AIDS/HIV Program. The project provided funding to HIV care sites for hiring additional support staff, called “linkage-to-care specialists,” to provide high-intensity case management and social support. Linkage-to-care specialists provided assistance beyond what is typically provided by medical case managers. For example, they helped participants sign up for government benefits, find housing, utilize the food pantry and other services offered at the clinic, and provided transportation to clinic appointments. Participants who reported no missed clinic appointments cited the assistance from their linkage-to-care specialist as the main reason they were able to stay in care, often due to the transportation assistance they received. One 45 year-old man estimated that 90% of the financial help he received post-incarceration came from his linkage-to-care specialist’s assistance. Similarly, a 26 year-old man, discussed how his relationship with his linkage-to-care specialist prior to the date of his release from prison helped build his skills and confidence to manage his HIV:

*I’m really glad that they brought [linkage-to-care specialist] into us, so now I have someone I can call and say, hey, I need to set up health insurance. Um, hey, I need to set up an appointment with dental… Once I know where to go with all this, once I’m educated on this, I can handle it myself… But I just need her to educate me.* (26 year-old man, pre-release interview)

## Discussion

In this analysis of semi-structured interviews among a cohort of people living with HIV who are released from prison, we found evidence supporting a pervasive impact of HIV stigma, which influences the way people seek and receive care during the re-entry period. Internalized stigma, in particular, was cited as an important barrier to engagement in HIV care upon release from prison by the majority of participants. The real and/or perceived social consequences of identifying as a person living with HIV seemed to affect nearly all of participants’ experiences navigating the health care system and the social interactions of their daily lives.

Our findings are consistent with previous conceptualizations of the role of HIV stigma in HIV care. Earnshaw and Chaudoir (2009) developed the HIV Stigma Framework to understand the mechanisms through which stigma works and its relevant consequences. The manifestations of stigma documented through our analysis mirror the mechanisms of HIV stigma in their proposed model, in which stigma can be “anticipated,” “enacted” (experienced) and “internalized” by people living with HIV, to the detriment of their physical health, mental health, and social relationships.

Our study adds new empirical data in support of prior efforts to document and measure the role of stigma in the lives of people living with HIV. The Internalized AIDS-Related Stigma Scale, developed by Kalichman et al. (2009), is a validated measure of participants’ identification with common internal representations of HIV, such as “being a bad person” or “unclean” (Kipp et al., 2015). The shame and fear of status disclosure has been linked to adverse mental health outcomes, psychological distress, and substance use, which are well-described barriers to HIV care (Alonzo & Reynolds, 1995; Earnshaw, Smith, Chaudoir, Amico, & Copenhaver, 2013; Pulerwitz, Michaelis, Weiss, Brown, & Mahendra, 2010; Small et al., 2009; Taylor, 2001).

Social support and increased social network size have been demonstrated in prior research to be resiliency factors against internalized stigma (Beals, Peplau, & Gable, 2009; Earnshaw, Lang, Lippitt, Jin, & Chaudoir, 2015; Logie & Gadalla, 2009; Sowell & Phillips, 2010). Participants in this study who received high levels of social support in the reintegration process reported greater success in medication adherence and fewer missed appointments with HIV care providers. Social support, especially from objective professional sources such as linkage-to-care specialists, served as an important resiliency factor that mitigated stigma and helped participants navigate both anticipated and unanticipated barriers to care upon release. A previous study evaluating the Wisconsin linkage-to-care specialist intervention found that social support provided by professional case managers helped to motivated and enable clients to adhere to HIV care, leading to improved outcomes such as undetectable viral load (Broaddus, Hanna, Schumann, & Meier, 2015).

Our findings have several implications for the delivery of HIV care to patients who were recently incarcerated. Sensitivity to the experiences of HIV stigma should inform the design of waiting room environments to provide greater privacy. When alteration of the clinic environment is not feasible, our study indicates that supportive relationships with staff and a welcoming social environment can ameliorate disclosure concerns among patients who attend clinic appointments. This is also supported by a recent study by Rozanova, Brown, Bhushan, Marcus, and Altice (2015), who demonstrated important benefits of establishing a trusting relationship between criminal justice-involved patients and their providers. In such trusting relationships, patients are more likely to be honest about their adherence and any challenges they face and providers will have a more realistic understanding of barriers that are modifiable and can be addressed (Joachim & Acorn, 2000; Vanable, Carey, Blair, & Littlewood, 2006). Clinical providers should also support patients by encouraging them to involve supportive family members or friends in their care, and to be aware of the level of social support an individual receives from their personal relationships (Aberg et al., 2014).

Our study has several important limitations. In-depth examination of HIV stigma was not specified as a research question in the original study protocol, but rather emerged as a common and influential barrier to care through the semi-structured interviews. As such, we did not incorporate existing validated measures of HIV stigma into the scheduled study assessments, which would have allowed us to more rigorously assess stigma among our participants and directly compare our findings to prior published work.

An additional limitation of the study was inherent in the convenience sampling method for participant recruitment. All participants interviewed resided in a single U.S. state, and the majority lived in a single large city after they were released. Their experiences may therefore be imperfectly representative of individuals who receive HIV care in other regions of the U.S. or in other countries. Racial disparities in incarceration and HIV are particularly severe in Wisconsin: One analysis indicated that 12.8% of the state’s African American adult male population was incarcerated at the time of the 2010 U.S. Census, compared to a national average of 6.7% (Pasawarat & Quinn, 2013). African Americans make up 44% of new HIV diagnoses in Wisconsin in 2014, but comprise less than 7% of the overall population (Wisconsin Department of Health Services, 2015). Our study sample, which was comprised predominantly of African American men, reflects the experience of a highly disadvantaged community of extraordinary public health importance.

Contrary to expectations, we did not find that participants’ descriptions of stigma were unique in the context of the pre-release versus the post-release interviews. The longitudinal study data did not illuminate, for example, instances of anticipated HIV stigma during the pre-release period, and enacted stigma during the post-release period. The reason for this was that nearly all participants had been incarcerated and released multiple times in the past, and had experienced relevant transitions in HIV care prior to enrollment in the study. Finally, the “linkage to care specialist” intervention occurring in Wisconsin during the study period provided a level of social and material support that is not typically available to most patients who require HIV care after release from prison. While this may limit the generalizability of our findings, our results suggest that similar interventions could play a beneficial role in overcoming HIV stigma and other barriers to care in diverse settings.

## Conclusions

HIV-related stigma is an important example of the numerous, complex psychosocial factors that can contribute to suboptimal engagement in HIV care for adults transitioning from prison to the general community. Stigma influences care-seeking behavior through internalization of negative attitudes about HIV and contributes to significant anxiety related to disclosure of HIV status. Individual-level interventions providing social support may mitigate the psychological distress associated with the manifestations of HIV stigma. Acknowledgment of HIV stigma in criminal justice settings should also inform clinic-level and systems-level change to enhance supportive care environments and reduce unnecessary barriers to care.

## Interview Guide

### Pre-release interview guide

1. Tell me a little bit about yourself.

-  How long have you been incarcerated? How much have you been able to keep in contact with friends and family while you’ve been incarcerated?

2. If you feel comfortable, will you share a little bit about how you found out that you were positive?

-  What did you do after hearing the news?
-  How were you feeling? Do you still feel that way now?
-  Did you tell anyone? If so, who did you share with?

3. Tell me about what it has been like for you to come to this clinic for HIV treatment.

-  What have you liked about receiving treatment at this clinic?
-  What haven’t you liked?
-  What has your doctor helped you with the most?

4. [If known positive before incarceration]

[If YES]

-  How would you describe your relationship with that provider?
-  What was the clinic/hospital like? How did it compare to your care at UW?
-  How frequently would you see your HIV doctor when you were living in the community?
-  Since the first time you saw a doctor about your HIV, what is the longest period of time you have gone without seeing a doctor for your HIV or getting your HIV labs done?

[If participant indicates a gap in care]

-  Thinking about this period of time, tell me more about what else was happening in your life.
-  Probe for more information about work, housing, incarceration, substance use, relationships, etc.
-  What was your biggest priority in your life at that time?
-  Did you feel healthy/well during those times?
-  What was it that got you back into regular care?

Are there any skills or strategies you have developed that help you keep coming in for regular HIV care visits?

5. Did you ever see a doctor or other provider for HIV care before you were incarcerated?
6. What makes a good HIV doctor? What is the most important characteristic about a HIV doctor, to you?
7. In general, what encourages you to keep coming in for regular care?

-  Once finding out your status, how long was it before you started HIV medication?
-  How many different HIV medications have you tried?
-  Have you experienced negative side effects?
-  What was the longest period you went without taking meds? What was going on during that time? How was your health during that time?
-  What motivates you to take your meds?
-  How will it be to take your HIV meds once you rejoin the community?

Are there skills or strategies you have developed to remember to take your meds? Will those work in the community? How so or why not?

8. Tell me about your experience taking medication for HIV.

-  Where are you heading to? Is that a place you’ve lived before?
-  Where will you stay?
-  What do you anticipate doing the first few days?

9. Tell me about your plans for release.

-  What are your top priorities?
-  What resources do you have to help you achieve those priorities?
-  Thinking about these other priorities in your life, how will these affect your HIV medical care? Why?
-  Do you have a place in mind where you plan to receive HIV care? Is that a place you have been before? How are you feeling about going there?
-  Do you have an appointment with an HIV provider in the community?
-  How will you get to that appointment?

10. Tell me about your priorities for your first few months.
11. If a clinic or community program was going to develop a program to help people stay in regular HIV care after they are released from prison, what would be the most helpful things for the program to offer? Why are these things the most important?
12. Our hope is that you will participate in the study for the next 6 months. That will involve a monthly call and a longer call at 6 months, where we’ll ask a lot of questions like we did today. Where do you expect to be in 6 months?

-  How will your health be?
-  Is there anything that you fear about the next 6 months?

### Post-Release Interview Guide

***Icebreaker/General reflection on first 6 months***

1. It has been about 6 months since you were released from prison. What have those 6 months been like for you?

-  What were your first few days like?

i.   Are you where you thought you’d be?

b.  What things were harder than you expected them to be? What things were easier?
c.  What has been the biggest success for you in the last 6 months?

i.   What has made you the happiest?
ii.   What has been the best thing that happened to you since you rejoined?

2. What did you find helpful during your transition?

-  Were there important people in your life that encouraged you to stay engaged in your HIV care?

i.   Did you meet those people after your release or did you know them before? What did they offer you?

b.  What have you needed that you have received?

i.   What do you wish you knew before leaving?

3. Some people we’ve talked to said there were a lot more challenges that they anticipated initially. What challenges do you suppose they experienced?

-  What challenges did you experience?

i.   Thinking about your life before you were incarcerated – your life while incarcerated – and today, how do they compare?

b.  Looking back and recalling what helped you or what brought challenge, if someone were to create a system to help people transition back to the community, what should that program offer?

i. *  How long should those things be available?*

***Transition to HIV care in community***

4. Tell me about your transition from getting treatment for HIV in prison, to getting HIV care in the community. How does this compare to what you expected it to be like?
5. [If participant attended an appointment with an HIV provider]

Tell me about the clinic where you went for HIV care. Was there anything about the clinic that made it easier for you to keep your appointment?

-  Were there things that made it hard to keep your appointments with your HIV provider? What helped you overcome these challenges?
-  How many scheduled appointments did you miss with your provider? Share with me why you were unable to make the appointment(s).
-  Describe your relationship with your HIV provider.
  [If participant did not attend appointment with HIV provider].
  What were the main reasons you did not see an HIV provider after you were released from prison? What things might encourage you to get back into care?

***Medication adherence post-release***

6. [If participant remained in care & continued ART]

Did you encounter any challenges that made it hard to stay on your medications?

-  What helped you overcome these challenges?
-  How many days did you miss your meds?

[If participant discontinued ART for 3 days or more].

Tell me about what was going on in your life at the time you stopped taking medications for HIV.

-  What were the main reasons you did not take your meds?
-  Is there anything you can think of that would have made it easier for you to keep taking your medications?

***General health/HIV knowledge***

7. How often do you think about your health, specifically HIV, these days as compared to 6 months ago?

-  How confident are you that you can manage your HIV care for the next year? On a scale of 1 to 10, 1 being “not confident at all” and 10 being “totally confident”. Where would you put yourself?
-  Do you recall your most recent CD4 count and viral load?
-  If problems with your health in the future, what will you do?

i.   How does your health impact your life?

8. [If applicable]
  Upon your release, you were connected to a linkage to care specialist [insert specialists name here]. Describe your relationship with them. In what ways were they helpful? Did you find them to be unhelpful?
9. [If applicable]

You spoke a few times about your parole officer. Please describe your relationship with them. In what ways were they helpful to you? Did you find them to be unhelpful? Does your parole officer know your HIV positive?

***Role of study***

10. What has it been like for you to be part of this study?

-  How would your first 6 months have been if you were not doing the study?

i.   How would the first 6 months be different for people who did not participate in the study?

b.  [If used study cell]
c.  Did you use your study cell phone for reasons other than the study?
d.  How are you feeling about completing the study today?

## Abbreviations

AIDS: acquired immunodeficiency syndrome
ART: antiretroviral therapy
DOC: Department of Corrections
HIV: human immunodeficiency virus
MSM: men who have sex with men
sIMB: situated information, motivation, and behavioral skills model

## References

[CR1] Aberg JA, Gallant JE, Ghanem KG, Emmanuel P, Zingman BS, Horberg MA, Infectious Diseases Society of, A (2014). Primary care guidelines for the management of persons infected with HIV: 2013 update by the HIV medicine association of the Infectious Diseases Society of America. Clinical Infectious Diseases.

[CR2] Alonzo AA, Reynolds NR (1995). Stigma, HIV and AIDS: An exploration and elaboration of a stigma trajectory. Social Science & Medicine.

[CR3] Amico KR (2011). A situated-information motivation behavioral skills model of care initiation and maintenance (sIMB-CIM): An IMB model based approach to understanding and intervening in engagement in care for chronic medical conditions. Journal of Health Psychology.

[CR4] Baillargeon J, Giordano TP, Rich JD, Wu ZH, Wells K, Pollock BH, Paar DP (2009). Accessing antiretroviral therapy following release from prison. Jama.

[CR5] Beals KP, Peplau LA, Gable SL (2009). Stigma management and well-being: The role of perceived social support, emotional processing, and suppression. Personality and Social Psychology Bulletin.

[CR6] Brinkley-Rubinstein L (2013). Incarceration as a catalyst for worsening health. Health & Justice.

[CR7] Brinkley-Rubinstein L (2015). Understanding the effects of multiple stigmas among formerly incarcerated HIV-positive African American men. AIDS Education and Prevention.

[CR8] Broaddus MR, Hanna CR, Schumann C, Meier A (2015). "she makes me feel that I'm not alone": Linkage to care specialists provide social support to people living with HIV. AIDS Care.

[CR9] Churcher S (2013). Stigma related to HIV and AIDS as a barrier to accessing health care in Thailand: A review of recent literature. WHO South-East Asia Journal of Public Health.

[CR10] Earnshaw VA, Chaudoir SR (2009). From conceptualizing to measuring HIV stigma: A review of HIV stigma mechanism measures. AIDS and Behavior.

[CR11] Earnshaw VA, Lang SM, Lippitt M, Jin H, Chaudoir SR (2015). HIV stigma and physical health symptoms: Do social support, adaptive coping, and/or identity centrality act as resilience resources?. AIDS and Behavior.

[CR12] Earnshaw VA, Smith LR, Chaudoir SR, Amico KR, Copenhaver MM (2013). HIV stigma mechanisms and well-being among PLWH: A test of the HIV stigma framework. AIDS and Behavior.

[CR13] Goffman E (1963). Stigma: Notes on the management of spoiled identity.

[CR14] Haley DF, Golin CE, Farel CE, Wohl DA, Scheyett AM, Garrett JJ (2014). Multilevel challenges to engagement in HIV care after prison release: A theory-informed qualitative study comparing prisoners' perspectives before and after community reentry. BMC Public Health.

[CR15] Hsieh HF, Shannon SE (2005). Three approaches to qualitative content analysis. Qualitative Health Research.

[CR16] Joachim G, Acorn S (2000). Living with chronic illness: The interface of stigma and normalization. The Canadian Journal of Nursing Research.

[CR17] Kalichman SC, Simbayi LC, Cloete A, Mthembu PP, Mkhonta RN, Ginindza T (2009). Measuring AIDS stigmas in people living with HIV/AIDS: The internalized AIDS-related stigma scale. AIDS Care.

[CR18] Katz IT, Ryu AE, Onuegbu AG, Psaros C, Weiser SD, Bangsberg DR, Tsai AC (2013). Impact of HIV-related stigma on treatment adherence: Systematic review and meta-synthesis. Journal of the International AIDS Society.

[CR19] Kipp AM, Audet CM, Earnshaw VA, Owens J, McGowan CC, Wallston KA (2015). Re-validation of the van Rie HIV/AIDS-related stigma scale for use with people living with HIV in the United States. PloS One.

[CR20] Logie C, Gadalla TM (2009). Meta-analysis of health and demographic correlates of stigma towards people living with HIV. AIDS Care.

[CR21] Maruschak, L. (2012). *HIV in prisons, 2001–2010*. Washington, D.C.: Bureau of Justice Statistics Retrieved from https://www.bjs.gov/content/pub/pdf/hivp10.pdf.

[CR22] Meyer, J. P., Cepeda, J., Springer, S. A., Wu, J., Trestman, R. L., & Altice, F. L. (2014a). HIV in people reincarcerated in Connecticut prisons and jails: An observational cohort study. *Lancet HIV, 1*(2), e77–e84. doi:10.1016/S2352-3018(14)70022-0. 10.1016/S2352-3018(14)70022-0 PMC4249702 25473651

[CR23] Morse J (1995). The significance of saturation. Qualitative Health Research.

[CR24] Parker R, Aggleton P (2003). HIV and AIDS-related stigma and discrimination: A conceptual framework and implications for action. Social Science & Medicine.

[CR25] Pasawarat J, Quinn LM (2013). Wisconsin’s mass incarceration of African American males: Workforce challenges for 2013.

[CR26] Pulerwitz J, Michaelis A, Weiss E, Brown L, Mahendra V (2010). Reducing HIV-related stigma: Lessons learned from horizons research and programs. Public Health Reports.

[CR27] Rozanova J, Brown SE, Bhushan A, Marcus R, Altice FL (2015). Effect of social relationships on antiretroviral medication adherence for people living with HIV and substance use disorders and transitioning from prison. Health Justice.

[CR28] Small W, Wood E, Betteridge G, Montaner J, Kerr T (2009). The impact of incarceration upon adherence to HIV treatment among HIV-positive injection drug users: A qualitative study. AIDS Care.

[CR29] Sowell RL, Phillips KD (2010). Understanding and responding to HIV/AIDS stigma and disclosure: An international challenge for mental health nurses. Issues in Mental Health Nursing.

[CR30] Springer SA, Friedland GH, Doros G, Pesanti E, Altice FL (2007). Antiretroviral treatment regimen outcomes among HIV-infected prisoners. HIV Clinical Trials.

[CR31] Springer SA, Pesanti E, Hodges J, Macura T, Doros G, Altice FL (2004). Effectiveness of antiretroviral therapy among HIV-infected prisoners: Reincarceration and the lack of sustained benefit after release to the community. Clinical Infectious Diseases.

[CR32] Taylor B (2001). HIV, stigma and health: Integration of theoretical concepts and the lived experiences of individuals. Journal of Advanced Nursing.

[CR33] Vanable PA, Carey MP, Blair DC, Littlewood RA (2006). Impact of HIV-related stigma on health behaviors and psychological adjustment among HIV-positive men and women. AIDS and Behavior.

[CR34] Wisconsin Department of Health Services (2015). Wisconsin HIV/AIDS surveillance annual report.

